# Snails in the desert: Species diversification of *Theba* (Gastropoda: Helicidae) along the Atlantic coast of NW Africa

**DOI:** 10.1002/ece3.3138

**Published:** 2017-06-22

**Authors:** Carola Greve, Martin Haase, Rainer Hutterer, Dennis Rödder, Flora Ihlow, Bernhard Misof

**Affiliations:** ^1^ Stiftung Zoologisches Forschungsmuseum Alexander Koenig Bonn Germany; ^2^ Vogelwarte, Zoologisches Institut und Museum Ernst‐Moritz‐Arndt‐Universität Greifswald Greifswald Germany

**Keywords:** AFLP, COI, drift, ecological speciation, environmental niche analysis, geometric morphometrics, mitochondrial introgression, selection, *Structure* analysis, *Theba*

## Abstract

The spatial subdivision of species often plays a pivotal role in speciation. Across their entire range, species are rarely panmictic and crucial consequences of spatial subdivision are (1) random genetic drift including historical factors, (2) uniform selection, and (3) divergent selection. Each of these consequences may result in geographic variation and eventually reproductive isolation, but their relative importance in speciation is still unclear. In this study, we used a combination of genetic, morphological, and climatic data to obtain a comprehensive picture of differentiation among three closely related, parapatrically distributed taxa of the land snail genus *Theba* occurring along the Atlantic coasts of South Morocco and Western Sahara. We conducted Mantel and partial Mantel tests to relate phenotypic and genotypic variation of these species to geography and/or climate. As null hypothesis for an evolutionary scenario, we assumed nonadaptive speciation and expected a pattern of isolation by distance among taxa. Rejection of the null hypothesis would indicate isolation by environment due to adaptation. Generally, genetic drift plays an important role but is rarely considered as sole driver of speciation. It is the combination of drift and selection that predominantly drives speciation. This study, however, provides a potential example, in which nonadaptive speciation, that is, genetic drift, is apparently the main driver of shaping the diversity of *Theba* in NW Africa. Restriction of gene flow between populations caused by geographic isolation probably has played an important role. Climate oscillations during the Plio‐ and Pleistocene may have led to repeated ecological changes in NW Africa and disruptions of habitats promoting differentiation by geographic isolation. The inferred evolutionary scenario, however, did not fully explain the incongruence between the AFLP‐ and mtDNA‐tree topologies. This incongruence might indicate past hybridization among the studied *Theba* forms.

## 1 INTRODUCTION

The spatial subdivision of species ranges often plays a pivotal role in speciation. Natural populations are rarely panmictic, and crucial consequences of spatial subdivision are (1) random genetic drift including historical factors, (2) uniform selection acting on separate subpopulations experiencing similar selection regimes, and (3) divergent selection acting on separate subpopulations living in different ecological environments. Each of these consequences may result in geographic variation and eventually reproductive isolation, but their relative importance in speciation is still unclear and probably case dependent (Coyne & Orr, 2004; Dieckmann, Doebeli, Metz, & Tautz, 2004; Gavrilet, 2003; Schluter, 2000, 2009).

Due to their low vagility, the population structure of land snails is often composed of many local subpopulations experiencing little genetic contact (Thomaz, Guiller, & Clarke, 1996). Speciation in land snails thus could be assumed to be largely driven by allopatry and genetic drift as opposed to natural selection (Cook, 2008; Davison, 2002; Davison & Clarke, 2000; Goodacre, 2001; Greve, Gimnich, Hutterer, Misof, & Haase, 2012; Holland & Hadfield, 2004; Sauer, Oldeland, & Hausdorf, 2013; Scheel & Hausdorf, 2012). However, genetic drift is rarely considered as sole driver of speciation. It is the combination of drift and selection that predominantly drives species divergence (Marie Curie Speciation Network, 2012).

Here, we investigated the spatial differentiation between three closely related, parapatrically distributed taxa of the land snail genus *Theba* Risso, 1826 occurring along the Atlantic coasts of South Morocco and the Western Sahara.

Today, the coast of the Western Sahara is characterized by an arid climate with less than 50 mm mean annual rainfall, whereas the coastline of South Morocco is more humid with up to 100 mm mean annual rainfall (Hijmans, Cameron, Parra, Jones, & Jarvis, 2005; Monod, 1958). The whole region, however, was affected by major ecological changes due to periodical climate oscillations during the Pliocene‐Pleistocene interval (the last ca. 5.3 million years; DeMenocal, 2004). The climatic instability led to periodic modifications of the ecological system, including expansion/contraction of the Saharan desert (Anhuf, 2000; Rognon, 1993), which in turn entailed new selection regimes and/or geographical isolation potentially leading to genetic differentiation of populations, adaptation, and ultimately speciation.

**Figure 1:**
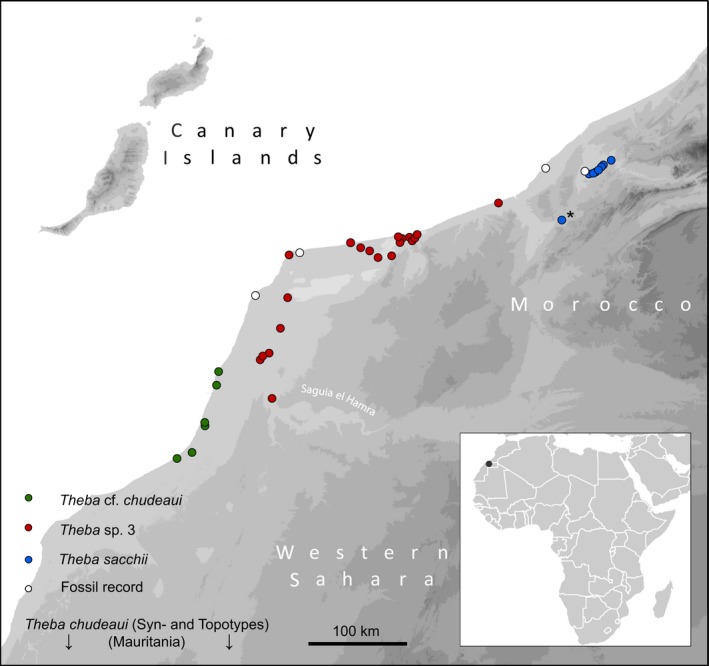
Sampling localities of specimens of *Theba* cf. *chudeaui*,* Theba sacchii,* and *Theba* sp. 3 along the Atlantic coast of South Morocco and Western Sahara. * The only sampling locality, where we found living animals of *T. sacchii*

Species of *Theba* occur on shrubs scattered on rocky plains and sand dunes. *Theba* sp. 3 and *T*. cf. *chudeaui* inhabit a narrow coastal strip of South Morocco and the Western Sahara. Syn‐ and topotypes of *T. chudeaui* were collected at Cap Blanc (=Ras Nouadhibou) in Mauritania, which is about 850 km further south. *Theba* sp. 3 and *T*. cf. *chudeaui*, however, are separated by a deep valley (Saguia el Hamra), which is occasionally flooded after heavy rainfall. The shell morphologies of both taxa are very similar and only molecular data recently identified them as two genetically distinct lineages (Greve, Hutterer, Groh, Haase, & Misof, 2010; Haase, Greve, Hutterer, & Misof, 2014). The third species, *T. sacchii*, has a morphologically distinct shell shape and is found further north in more inland areas of South Morocco (Figure 1, Table 1 and Table S1).

**Table 1:** Summary of specimens used in the present study

Species	Code	No. of specimens genotyped (AFLP)	No. of specimens used in morphometrics	COI GenBank accession #
*Theba subdentata*	M24	3	–	HM034526, HM034528, KC526931
*Theba subdentata*	M55	2	–	HM034529
*Theba solimae*	M25	3	9	HM034494, KC526932–526933
*Theba solimae*	M27	1		HM034495
*Theba solimae*	M59	–	6	–
*Theba solimae*	M60	–	9	–
*Theba* sp. 3 large	M28	11	20	HM034473–034474, KC526941
*Theba* sp. 3 large	M36	2	35	HM034480
*Theba* sp. 3 large	M69	–	4	–
*Theba* sp. 3 large	M70	–	14	–
*Theba* sp. 3 large	M71	–	19	‐
*Theba* sp. 3 large	M72	–	8	–
*Theba* sp. 3 small	M33/48	3	10	HM034483, KC526942
*Theba* sp. 3 small	M34	9	6	HM034475–034477, KC526943
*Theba* sp. 3 small	M35/37/56	21	5	HM034478–034479, HM034481–034482, HM034484, KC526944–526947
*Theba* sp. 3 small	M73	–	5	‐
*Theba* sp. 3 small	M74	–	15	–
*Theba* sp. 3 small	M75	–	15	–
*Theba* sp. 3 small	M76	‐	17	–
*Theba* sp. 3 small	M77	–	19	–
*Theba* sp. 3 small	M78	‐	14	–
*Theba* sp. 3 small	M79	–	3	–
*Theba sacchii*	M39	14	13	HM034472, KC526937–526940
*Theba sacchii*	M61	‐	9	–
*Theba sacchii* (Holotype)	M62	–	1	–
*Theba sacchii*	M63	–	18	‐
*Theba sacchii*	M64	–	2	–
*Theba chudeaui* (Syn‐ and Topotypes)	M82	–	19	‐
*Theba* cf. *chudeaui*	M29	1	13	HM034485
*Theba* cf *chudeaui*	M30	4	6	HM034486–034487
*Theba* cf. *chudeaui*	M31	1	21	HM034488
*Theba* cf. *chudeaui*	M32	10	14	HM034489–034491, KC526934–526936
*Theba* cf. *chudeaui*	M80	–	2	–
*Theba* cf. *chudeaui*	M81	–	7	–
‘Fossil’	M65	‐	12	–
‘Fossil’	M66	–	32	–
‘Fossil’	M67	–	34	–
‘Fossil’	M68	–	5	–

For more details, see Table S1. Records of species, which were only used for environmental niche analyses, are listed in Table S1.

This suggested that diversification among these forms along the Atlantic coast has probably been driven by a mixture of nonadaptive and adaptive speciation. In case of *T. sacchii*, we assumed that natural divergent selection has led to morphological and ecological divergence, while genetic drift including historical separation by past climate oscillations may explain the morphological similarity of *Theba* sp. 3 and *T*. cf. *chudeaui*.

Herein, we used a combination of genetic, morphological and ecological criteria to obtain a comprehensive picture of differentiation and to identify the drivers of speciation: (1) AFLP markers were generated and mitochondrial DNA sequenced to resolve phylogenetic relationships and to investigate the genetic differentiation between the three Moroccan/Western Saharan taxa *Theba* sp. 3, *T*. cf. *chudeaui* and *T. sacchii*; (2) geometric morphometric (Zelditch, Swiderski, Sheets, & Fink, 2004) comparisons of shell shape were performed in order to complement the picture of differentiation of these partially cryptic forms; (3) the potential geographical distribution of each lineage was estimated based on environmental/climatic data (Elith & Leathwick, 2009); and (4) ecological niche differentiation among these taxa was assessed using a multivariate niche overlap analysis (Blonder, Lamanna, Violle, & Enquist, 2014).

As null hypothesis for an evolutionary scenario, we assumed nonadaptive speciation and expected a pattern of isolation by distance (IBD) among taxa, that is, a linear relationship between genetic and morphological distances on the one hand with geographic distances on the other. The environment, that is, climate, should have no influence (Bohonak, 1999; Orsini, Vanoverbeke, Swillen, Mergeay, & Meester, 2013; Wright, 1943). Rejection of the null hypothesis would indicate isolation by environment (IBE) due to adaptation, which is assumed to be most often responsible for phenotypic and ecological differentiation among species (Orr, 1998; Rundell & Price, 2009; Schluter, 2009).

## 2 MATERIALS AND METHODS

### 2.1 Sampling and DNA isolation

Snails of *Theba* sp. 3, *T*. cf. *chudeaui,* and *T. sacchii* were collected from 10 localities along the Atlantic coast of South Morocco and the Western Sahara in March 2008 (Figure 1; Table 1 and Table S1). Additionally, specimens of *T. solimae* and *T. subdentata* were collected in West Morocco (Table 1 and Table S1), the latter as outgroup species. Snails were preserved in absolute ethanol. Total genomic DNA was extracted from tissue samples from the foot muscle using the DNeasy^®^ Blood & Tissue Kit of Qiagen.

### 2.2 Mitochondrial DNA sequencing

At least one specimen per locality was sequenced for a fragment of the mitochondrial cytochrome *c* oxidase subunit I (COI). The COI fragment was amplified by the polymerase chain reaction (PCR) using the primer combination LCO‐1490 [5′GGTCAACAAATCATAAAGATATTGG‐3′ (Folmer, Black, Hoeh, Lutz, & Vrijenhoek, 1994)] and C1‐N‐2191 [5′‐CCCGGTAAAATTAAAATATAAACTTC‐3′ (Simon et al., 1994)]. PCR reactions were carried out in a total volume of 10 μl using the Qiagen Multiplex PCR Kit. Thermal cycling conditions were as follows: 95°C for 15 min, 15 cycles of touchdown PCR (94°C for 35 s, 55°C–40°C annealing for 90 s and 72°C extension for 90 s) followed by 25 cycles (94°C for 35 s, 40°C annealing for 90 s and 72°C extension for 90 s) and a final extension step at 72°C for 10 min. PCR products were purified using ExoSAP‐IT^®^ (USB). Both strands were sequenced on an ABI 3130xl Genetic Analyser (Applied Biosystems) using the BigDye^®^ Terminator v3.1 Cycle sequencing Kit (Applied Biosystems). Sequences were deposited in GenBank (Table 1 and Table S1).

### 2.3 AFLP genotyping

AFLP markers were obtained with a slightly modified version of the original protocol of Vos et al. (1995). Selective amplifications were performed using six different primer combinations: *Eco*RI‐ACA/MseI‐CTG, *Eco*RI‐ACA/MseI‐CTT, *Eco*RI‐ACC/MseI‐CAC, *Eco*RI‐AGG/MseI‐CTG, *Eco*RI‐AGG/MseI‐CTC and *Eco*RI‐ACT/MseI‐CAG. The fluorescently labeled fragments were separated by electrophoresis on a CEQ™ 8800 capillary sequencer (Beckman Coulter, Inc., Fullerton, California), with an internal size standard (CEQ DNA Size Standard Kit 600, Beckman Coulter, Inc.). Signal detection, processing, and binning of the AFLP electropherograms were carried out using the CEQ™ System Fragment Analysis v. 9.0.25 (Beckman Coulter). The fluorescence threshold for an accepted signal was set to 1% of the height of the second largest peak detected in the AFLP profile. Correct fit of the size standard and fragment distribution was checked for all profiles. Low quality profiles were discarded. Subsequently, fixed fragment categories (hereafter also referred to as bin) were created between 55 and 550 bases (b). AFLP markers were automatically scored according to the presence/absence of fragment peaks within each bin and for each sample. The fluorescent signal detection threshold was set to 100 units for bins created between 55 and 299 b and to 50 units for bins created between 300 and 550 b. According to the accuracy of the CEQ sequencing system (standard deviation = 0.25 b; manufacturer's specifications), the maximum bin width for reliable fragment sizing was set to 0.75 b. Monomorphic markers were excluded from the data set.

To ensure high reliability of AFLP genotyping, 22% of the samples were genotyped twice (hereafter also referred to as replicates) for all primer combinations; these replicates were taxonomically representative for the whole data set. After AFLP profiles had been evaluated by commercial genotyping software (such as CEQ™ System Fragment Analysis v. 9.0.25, Beckman Coulter), an automated scoring approach, called AMARE (AFLP MAtrix REduction; Kück, Greve, Misof, & Gimnich, 2012), was used for subsequent AFLP marker selection. Based on replicates, AMARE estimates the repeatability of each individual marker to control for scoring errors. The present AFLP character matrix was generated by the following parameter settings of AMARE: Bin reliability (BR) = 0.9, replicate reliability (RR) = 0.8, and bin distance (BD) = 0.0. Finally, the remaining markers were used to estimate the average genotyping error rate per marker (following Bonin et al., 2004; Pompanon, Bonin, Bellemain, & Taberlet, 2005).

### 2.4 Phylogenetic analyses

Phylogenetic reconstruction based on the AFLP data set was performed with PAUP* v4.0b10 (Swofford, 2002) using neighbor joining (NJ) on Nei‐Li (Nei & Li, 1979) distances. This distance measure is very well suited for AFLPs, as it accounts for the sharing of present alleles, while shared absent alleles are ignored due to their potential homoplasious character (Dasmahapatra, Hoffman, & Amos, 2009; Koopman, 2005). Internal node support was assessed by nonparametric bootstrapping (1,000 replicates).

COI sequences were aligned with ClustalW (Thompson, Gibson, & Higgins, 2002) using default parameter settings, and obviously misaligned positions were adjusted manually in Bioedit v7.0 (Hall, 1999). Homogeneity of base frequencies among COI sequences was checked with the χ^2^ ‐ test implemented in PAUP* v4.0b10 (Swofford, 2002). According to the results of the Akaike Information Criterion in MrModeltest v2.3 (Nylander, 2004), the GTR + Γ + I model was selected as the best model of sequence evolution. Based on the selected model, phylogenetic reconstruction was performed with MrBayes v3.1 (Huelsenbeck & Ronquist, 2001; Ronquist & Huelsenbeck, 2003). The Bayesian analysis consisted of two runs each with four simultaneous Markov chains for 20,000,000 generations. Trees were sampled every 100th generation. Excluding the first 50,000 trees of each run as burn‐in, a 50% majority‐rule consensus tree was constructed. Posterior probabilities were calculated based on the remaining 300,002 trees. Tracer v1.4.1 (Rambaut & Drummond, 2008) was used to determine the burn‐in generation number as well as to check convergence of parameter estimates by inspecting effective sample size (ESS) values and traces of the MCMC samples.

### 2.5 Structure

*Structure v*2.3.2 (Falush, Stephens, & Pritchard, 2007; Pritchard, Stephens, & Donnelly, 2000) was used to investigate patterns of genetic structure, based on the AFLP data set. Analyses were conducted without a priori group designation using a model allowing for recessive alleles, which is best suited for dominant molecular markers such as AFLPs (Falush et al., 2007). We chose an admixture model with correlated allele frequencies (Falush, Stephens, & Pritchard, 2003). We allowed for gene flow, thus avoiding potentially inaccurate presumptions about genetic barriers. The Dirichlet parameter for the degree of admixture (α) and the parameter of allelic frequency distribution (λ) were set to be inferred from the data. For all *Structure* analyses, we used a total run length of 250,000 generations, including a burn‐in of 50,000 generations. According to the number of sampled populations, *K *=* *1 to 14 was tested with ten independent runs at each *K* (=number of populations or clusters). We plotted the mean likelihood *L*(*K*) over 10 runs for each *K* and used the statistic Δ*K* proposed by Evanno, Regnaut, and Goudet (2005) to determine the optimal number of genetically differentiated clusters. The graphical display of the *Structure* results was generated using *Distruct* software (Rosenberg, 2002).

### 2.6 Morphometrics

The morphometric comparisons also included syn‐ and topotypes of *T. chudeaui* from Mauritania and fossils previously ascribed to *T. chudeaui* (Hutterer, Greve, & Haase, 2010), thus all taxa involved in this complex of partially cryptic species. In total, 447 adult shells (Table 1 and Table S1) were subjected to geometric morphometrics in order to analyze shape and size separately (Zelditch et al., 2004) following the procedure of Haase and Misof (2009). Shells were photographed in apertural view, all at the same scale. Ten landmarks (see Figure 5) were placed using the programs tpsUtil (Rohlf, 2004a) and tpsDig 2.0 (Rohlf, 2004b). All subsequent analyses were conducted in version 6 of the IMP suite of programs by Sheets and colleagues (http://www3.canisius.edu/sheets/morphsoft.html) as well as with PAST 2.0 (Hammer, Harper, & Ryan, 2001) and based on partial Procrustes superimpositions. Multivariate regression of partial warp and uniform deformation scores on centroid size, which was used as a proxy for shell size, indicated allometry (Wilk's λ = 0.48, *F* = 29.21, *df*_1_ = 16, df_2_ = 430, variance explained = 52%, *p* < .001). The variance partition explaining size was then removed after regression on the centroid size of the smallest individual. The resulting standardized shape variables were submitted to a MANOVA/CVA followed by pairwise post hoc Hotelling's *T* ^2^ tests with four constraints. These comparisons were validated by CVA‐based assignment tests. Pairwise comparisons were also illustrated by thin‐plate splines.

### 2.7 Data acquisition and preparation for environmental niche analyses

N‐dimensional hypervolumes were performed based on locality records and a set of satellite‐derived environmental predictors. Records of *T. sacchii*,* T*. cf. *chudeaui,* and *Theba* sp. 3 were compiled from our own fieldwork and specimens housed in the National Museum of Natural History (formerly Rijksmuseum van Natuurlijke Historie), Leiden, the Netherlands. For *T. sacchi*, we also included records found in Gittenberger and Ripken (1987), as the shell shape of *T. sacchi* is morphologically clearly identifiable (see Table 1 and Table S1). In those cases where no coordinates but exact locality names were available, records have been georeferenced using google earth. The final data set comprised eight records for *T. sacchii*, six records for *T*. cf. *chudeaui,* and 16 records for *Theba* sp. 3. Reliability of all records was assessed by mapping them in DIVA‐GIS 7.5 (Hijmans, Guarino et al., 2005; http://www.diva-gis.org) for visual inspection.

The environmental predictors combining both bioclimatic variables acquired from the WorldClim database (Hijmans, Cameron et al., 2005) and preprocessed remote sensing variables were processed using the *raster* and *dismo* packages for *Cran R* (Hijmans & Van Etten, 2012; Hijmans, Phillips, Leathwick, & Elith, 2012; R Development Core Team, 2012). The remote sensing variables were derived from MODIS sensors of two NASA satellites (spatial resolution = 30 arcsec; temporal resolutions: (MOD11A2) = 8‐day averages; (MCD43B4) = 16‐day averages; Mu, Heinsch, Zhao, & Running, 2007; Scharlemann et al., 2008) available through the EDENext (2013) portal. This imagery collected from 2001 to 2005 was produced by the TALA Research Group, Oxford University using the methods described in Scharlemann et al. (2008). The data set contains the following variables: (1) middle infra‐red coding for water content within vegetation, (2) the normalized vegetation index (NDVI), (3) the enhanced vegetation index (EVI), and (4) monthly averages of day and night time land surface temperatures. Based on monthly averages of the remote sensing variables, we computed predictors analogue to the bioclimatic variables available from WorldClim (Beaumont, Hughes, & Poulsen, 2005; Nix, 1986). These derived predictors comprised annual averages (conceptually equivalent to bio1), average scores of the quarter with the highest and lowest scores (equivalent to bio10 and bio11), and the annual range of the respective variable (equivalent to bio7). In addition, we obtained the following bioclimatic variables from WorldClim (http://www.worldclim.org/): annual temperature and precipitation (bio1 and bio12), annual temperature range (bio7), mean temperature of the warmest quarter (bio10), mean temperature of the coldest quarter (bio11), precipitation of the wettest quarter (bio16), and precipitation of the driest quarter (bio17).

All predictor variables were resampled to the same resolution of 30 arcsec (~1 km) and clipped to the extent of the study area. In order to create an orthogonal environmental space, we randomly selected 10,000 localities throughout the study area, extracted the environmental conditions, and used these conditions to perform a principal component analyses in *Cran R*. All principal components with eigenvalues >1 were subsequently projected into geographic space.

### 2.8 Environmental niche comparisons via n‐dimensional hypervolumes

Following Hutchinson's niche concept (Hutchinson, 1957; Soberón, 2007; Soberón & Peterson, 2005), the realized environmental niche of a species is a subset of its fundamental niche reflecting all environmental conditions which support infinite existence of populations in the absence of biotic interactions. Using the orthogonal environmental space encompassed by the respective PCs, we characterized the realized niche of the (sub‐)species by computing n‐dimensional hypervolumes following Blonder et al. (2014). This technique extends the framework proposed by Broennimann et al. (2012) by applying multidimensional kernel density estimators to derive a density distribution of species records in PC space. This density distribution was subsequently used to compute the total volume of the realized niche space of each (sub‐)species by applying multivariate minimum convex polytopes enclosing all species records in environmental space as well as the intersection and unique proportions of relevant volumes. The results facilitated quantification of niche space shared by all lineages as well as of the unique portion occupied by each taxon as suggested by Guisan, Petitpierre, Broennimann, Daehler, and Kueffer (2014). Niche overlap was estimated using the Sørensen index based on unique and shared hypervolumes. All computations were performed in *Cran R* using the relevant functions of the *hypervolume* package (Blonder, 2014). When species records are limited in comparison with the dimensionality of the environmental space, and no additional records can be obtained with reasonable efforts, volume estimates might be close to zero requiring reduction of PCs.

Thus, we applied two different strategies: (1) including all PCs with eigenvalues >1 (*n* = 4, see below) in the analyses, and (2) considering only the first three PCs to reduce dimensionality.

All hypervolumes were subsequently projected into geographic space to delimit the potential distribution of each taxon. The discrimination ability of the hypervolume projections was assessed using the area under the receiver operating characteristic curve (AUC; Swets, 1988) and the point–biserial correlation (COR; Elith et al., 2006). As environmental background for model testing, we selected the environmental conditions within a spatial buffer of 100 km enclosing all species records.

### 2.9 Comparative analyses

In order to infer evolutionary scenarios among the four focal (sub‐)species, we jointly analyzed genetic, geographic, morphological, and climatic data performing Mantel and partial Mantel tests in PASSaGE (Rosenberg & Anderson, 2011). However, our analyses were constrained by the unbalanced availability (genetics: only few live collected specimens) and resolution (climate) of data. Therefore, we had to analyze on the species level and could not conduct other approaches such as multivariate redundancy analyses (RDA; Riordan et al., 2016). The matrices compared were composed of Nei‐Li genetic distances (AFLP), geographic distances between the centroids of the ranges, squared Mahalanobis distances (shape), and Euclidean distances based on the 4 PCs used to compute hypervolume models, respectively.

## 3 RESULTS

### 3.1 Phylogenetic analyses

**Figure 2:**
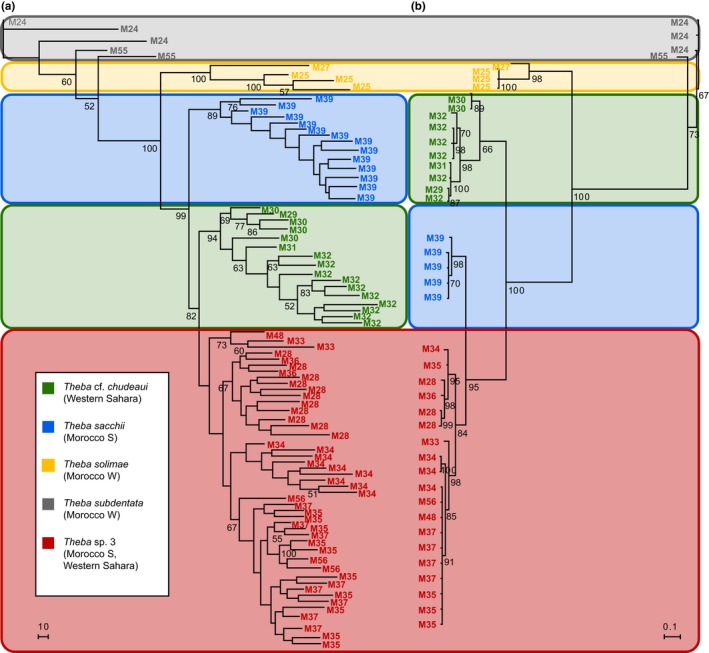
(a) AFLP neighbor‐joining (NJ) tree of *Theba* species from South Morocco and the Western Sahara based on Nei‐Li distances. The AFLP data set consisted of 767 loci. Bootstrap support values (1,000 replicates) are indicated below branches. (b) 50% majority‐rule consensus tree based on the Bayesian analysis of COI. Bayesian posterior probabilities (BPP) are indicated below branches

In total, 85 individuals were scored for 767 AFLP markers. The estimated genotyping error rate per marker was 2.7% and was lower than the maximum value of 10% recommended by Bonin, Ehrich, and Manel (2007). With *T. subdentata* as outgroup species, the result of the AFLP NJ tree analysis showed that *T. solimae* (bootstrap support (BS) = 100%) was sister to a monophyletic group (BS = 99%) comprising *T. sacchii* (BS = 89%), *T*. cf. *chudeaui* (BS = 94%), and *Theba* sp. 3 (BS < 50%). Within this group, *T*. cf. *chudeaui* and *Theba* sp. 3 were closely related (BS = 82%). Clades within *Theba* sp. 3 comprised individuals from identical sampling sites. In contrast to *T*. cf. *chudeaui* (BS = 94), monophyly of *Theba* sp. 3 was not supported (Figure 2a).

Forty‐two specimens were sequenced for COI resulting in a data set of 628 aligned positions. With a χ^2^‐value of 29.61 (*df* = 123) and *p *>* *.999, the hypothesis of homogeneous base composition among sequences was not rejected. In contrast to the AFLP NJ tree, COI data supported a sister group relationship between *T. sacchii* and *Theba* sp. 3 by a Bayesian posterior probability (BPP) of 95%. The monophyly of *T*. cf. *chudeaui* and *Theba* sp. 3 was only weakly supported (BPP = 66% and 84%, respectively; Figure 2b).

### 3.2 Structure

**Figure 3:**
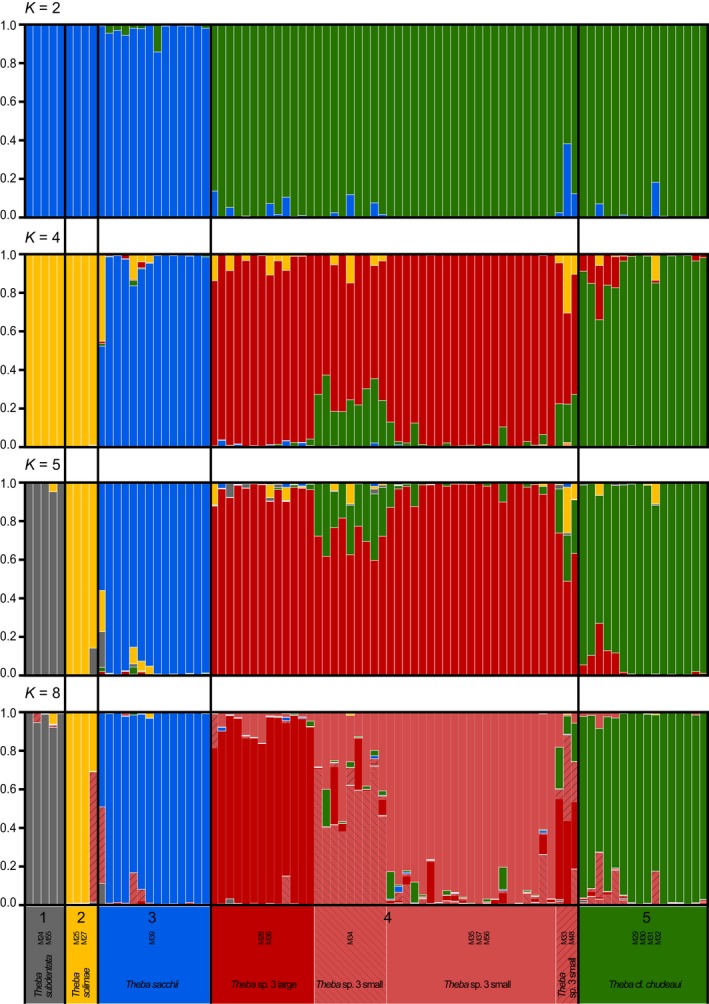
Results of the *Structure* analyses based on the AFLP data set. *Structure* graphical outputs from the run at each *K* with the highest likelihood for *K* = 2, *K* = 4, *K* = 5, and *K* = 8 are shown. Each individual is represented by a vertical bar colored in proportion to its estimated ancestry within each cluster. Numbers correspond to species division suggested by phylogenetic analyses: 1 = *T. subdentata*, 2 = *T. solimae*, 3 = *T. sacchii*, 4 = *Theba* sp. 3, 5 = *T*. cf. *chudeaui*

The results of the *Structure* analyses were consistent with the results of the phylogenetic analyses. The mean likelihood *L* (*K*) increased from *K* = 1 to a maximum value at *K* = 5 (−20,994.41) and then decreased to a minimum value at K = 13 (−31,898.99; Figure S1A). The statistics Δ*K* (Evanno et al., 2005) showed multiple peaks at *K* = 2 (Δ*K* = 144.32), *K* = 4 (Δ*K* = 78.11), *K* = 5 (Δ*K* = 7.53), and *K* = 8 (Δ*K* = 7.09; Figure S1B). At *K* = 2, individuals of *T. subdentata*,* T. solimae,* and *T. sacchii* grouped into one cluster, whereas *T*. cf. *chudeaui*, and *Theba* sp. 3 grouped into the other cluster. At *K* = 4, individuals of *T. sacchii, T*. cf. *chudeaui,* and *Theba* sp. 3 were each assigned to a separate cluster. The genetic differentiation suggested by the *Structure* analysis with the highest mean likelihood value (*K* = 5) corresponded to the species division suggested by the phylogenetic analyses of the present study: *T. subdentata*,* T. solimae*,* T*. cf. *chudeaui*,* T. sacchii,* and *Theba* sp. 3. Results of *K* = 8 showed additional genetic differentiation within *Theba* sp. 3, where specimens of *Theba* sp. 3 small (see section 3 of geometric morphometrics) grouped into separate genetic clusters in accordance with sampling sites and specimens of *Theba* sp. 3 large (see below results of geometric morphometrics) were assigned to a joint cluster (Figure 3).

### 3.3 Morphometrics

**Figure 4:**
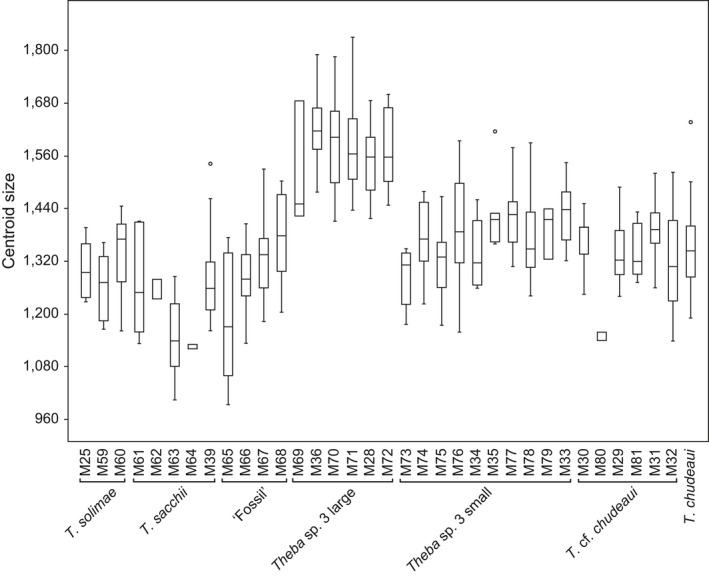
Box‐plots comparing centroid sizes. Samples roughly ordered from north to south (see Figure 1 and Table S1)

Based on morphological pilot studies (not shown) and the molecular analyses, we distinguished seven operational taxonomic units (OTUs) in our morphometric analyses: *T. solimae*,* T. sacchii*, ‘Fossil’, *Theba* sp. 3 large, *Theba* sp. 3 small, *T*. cf. *chudeaui*, and *T. chudeaui*. The differentiation within *Theba* sp. 3 partly coincided with the molecular *Structure* analysis assuming eight clusters (Figures 3, 4 and Figure S2). *Theba* sp. 3 small, which showed further signs of genetic subdivision, was morphologically homogeneous.

**Figure 5:**
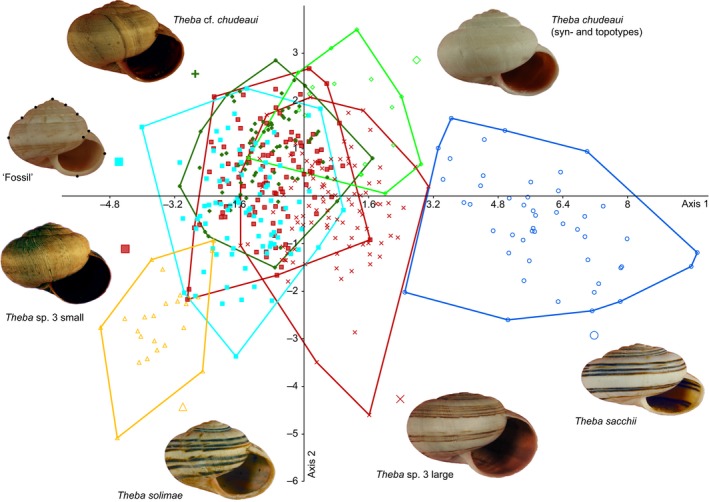
Canonical variates analysis based on 10 landmarks. Clockwise: cyan square, “Fossil,” with 10 landmarks; blue circle, *T. sacchii*; green diamond, *T. chudeaui* (syn‐ and topotypes); red cross, *Theba* sp. 3 large; red square, *Theba* sp. 3 small; yellow triangle, *T. solimae*; green plus, *T*. cf. *chudeaui*

In terms of size, all samples were in a similar range, only *Theba* sp. 3 large stood out (Figure 4). In a canonical variate analysis (CVA) comparing shell shape, *T. solimae* and *T. sacchii* were distinct, while the remaining OTUs overlapped to a large extent (Figure 5). However, pairwise Hotelling's *T* ^2^ tests distinguished all OTUs highly significantly (*p *<* *.001 in all cases after Bonferroni's correction) except the parapatric *Theba* sp. 3 small and *T*. cf. *chudeaui* (*p* = .118). This was also confirmed by the CVA‐based assignment test, in which all shells belonging to *T. sacchii* and *T. solimae*, respectively, were correctly classified and only 11.0% and 10.5% of shells of *Theba* sp. 3 large and *T. chudeaui*, respectively, incorrectly. The assignment of individuals belonging to *Theba* sp. 3 small and *T*. cf. *chudeaui* failed. In both OTUs, more than 50% of the shells were reciprocally misclassified.

Three pairwise shape comparisons were of particular interest, the first illustrating the differentiation within *Theba* sp. 3, the second contrasting “Fossil” with *Theba* sp. 3 small, to which they were most similar, and the third showing the differences between *T. chudeaui* from Mauritania and *T*. cf. *chudeaui* from Western Sahara (Figure S2). The smaller, southern form of *Theba* sp. 3 was more globular than the larger form. Specifically, the spire was more voluminous. “Fossil” was even more globular than *Theba* sp. 3 small, and *T*. cf. *chudeaui* was more conical than the shells from Mauritania.

### 3.4 Environmental niche comparisons via n‐dimensional hypervolumes

Contribution of each of the 23 predictors, comprising seven bioclimatic and 16 variables derived from remote sensing data, to each principal component is displayed inTable S2.

The first two principal components with eigenvalues of 12.01 and 4.09, respectively, explained 52.21% and 17.78% of the total variation among all predictors.

“Precipitation of the wettest quarter” (bio16) followed by “annual precipitation” (bio12) had the highest loadings on the first principal component (PC). The second PC was dominated by “precipitation of the driest quarter” (bio17) and “annual precipitation” (bio12). The third PC was mainly characterized by “mean temperature of coldest quarter” (bio11) and “mean temperature of coldest quarter (remote)” (ED150708_bio11) while “temperature annual range” (Bio 7) and “mean temperature of warmest quarter” (Bio 10) contributed most to the forth PC.

Model performance (AUC) for four‐dimensional hypervolumes for the three taxa of *Theba* varied between 0.992 for *T*. cf. *chudeaui* and 0.967 for *T. sacchii* demonstrating that the model discriminated well between suitable versus unsuitable space (Araujo & New, 2007; Phillips, Anderson, & Schapire, 2006; Swets, 1988). Within *Theba* sp. 3, the model also discriminated between *Theba* sp. 3 small (AUC: 0.967) and *Theba* sp. 3 large (AUC: 0.897). COR values were 0.484 for *T*. cf. *chudeaui*, 0.439 for *Theba* sp. 3 (with 0.594 for *Theba* sp. 3 large and 0.318 for *Theba* sp. 3 small) and 0.318 in *T. sacchii*.

However, when only the first three PCs were used to compute hypervolume models, model performances (AUC) were 0.967 for *T. sacchii,* 0.947 for *Theba* sp. 3, and 0.885 for *T*. cf. *chudeaui*. Within *Theba* sp. 3, the discrimination ability was 1.000 for *Theba* sp. 3 large and 0.946 for *Theba* sp. 3 small. COR values were 0.295 for *T*. cf. *chudeaui*, 0.311 for *T*. sp. 3, and 0.318 for *T. sacchii*. Within *Theba* sp. 3, COR values were 0.912 in *Theba* sp. 3 large and 0.248 in *Theba* sp. 3 small.

**Figure 6:**
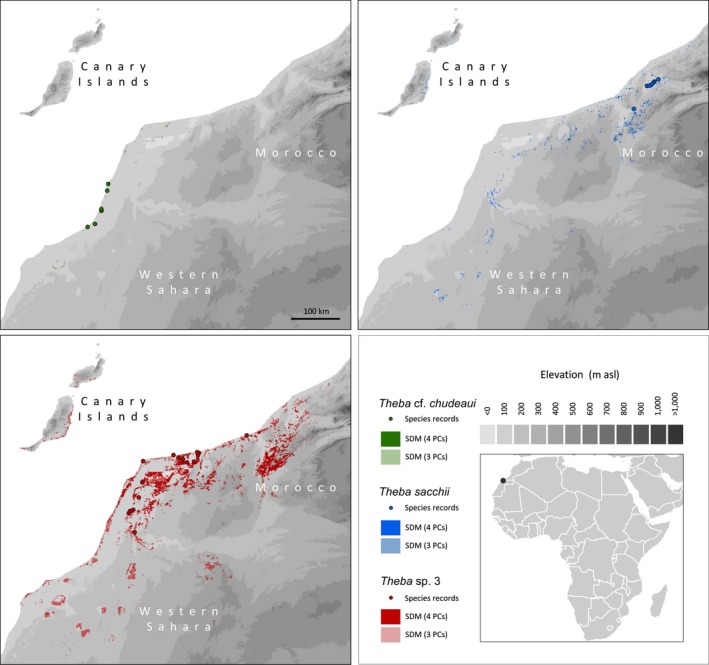
Potential distributions for *Theba* cf. *chudeaui*,* Theba sacchii,* and *Theba* sp. 3

The predicted potential distributions for all taxa of *Theba* were in concordance with the known distributional ranges (Figure 6). The spatial extent of the three‐dimensional hypervolume models was larger than the potential distribution predicted for the respective four‐dimensional hypervolume models. Thus, the three‐dimensional hypervolume model additionally highlighted potentially suitable space for *T*. *sacchii*,* T*. cf. *chudeaui,* and *Theba* sp. 3, including *Theba* sp. 3 small and *Theba* sp. 3 large, extending across the coastal areas of the islands of Fuerteventura and except for *T*. cf. *chudeaui* also Lanzarote.

In concordance with the known geographic distributions, the largest spatial extent was predicted for *Theba* sp. 3 (volume: 3PCs: 3.806; 4PCs: 1.413), followed by *T*. *sacchii* (3PCs: 0.228; 4PCs: 0.014) while the smallest range was predicted for *T*. cf. *chudeaui* (volume: 3PCs: 0.137; 4PCs: 0.003; Figures 1 and 6). As was to be expected, the potential distributions of *Theba* sp. 3 small (3PCs: 0.452; 4PCs: 0.109) and *Theba* sp. 3 large (3PCs: 0.217; 4PCs: 0.015) were nested within the range of the cluster *Theba* sp. 3 (Figure S3).

### 3.5 Niche overlap and intersection

The results of the multivariate niche overlap analyses (Figure S4) revealed low overlap between taxon‐pairs in terms of Sørensen Index between *T. sacchii* and *Theba* sp. 3 (3PCs: 0.037; 4PCs: 0.008) while no overlap was found between *T*. cf. *chudeaui* and any other taxon. In the nested group *Theba* sp. 3, no overlap was detected between *Theba* sp. 3 small and *Theba* sp. 3 large irrespective of the number of principal components used.

Intersection was only detected between *T. sacchii* and *Theba* sp. 3 (3PCs: 0.080; 4PCs: 0.006) *T*. cf. *chudeau*i did not overlap with any other taxon. In the nested group *Theba* sp. 3, no intersection was detected between *Theba* sp. 3 small and *Theba* sp. 3 large irrespective of the number of principal components used.

### 3.6 Comparative analyses

There were significant pairwise correlations between genetic and geographic distances (Mantel test: *r* = .817, *p* = .044), genetic and morphological (shell shape) distances (*r* = .812, *p* < .001), and morphological and geographic distances (*r* = .839, *p* < .001). Climate was uncorrelated to other data sets, although relationships with geography (*r* = −.634, *p* = .095) and morphology (*r* = −.595, *p* = .071) were close to significance. However, the relationships between morphology on the one hand and genetics as well as geography on the other hand disappeared when reciprocally controlling for confounding effects using partial Mantel tests (geography held constant: *r* = .401, *p* = .342; genetics held constant: *r* = .522, *p* = .197). Comparing morphology with climate controlling for geography also drastically lowered the absolute value of the correlation coefficient (*r* = −.151, *p* = .519), whereas the influence of climate on the relationship of morphology and geography was much smaller (*r* = .743, *p* = .096). In conclusion, there is 1) evidence for genetic isolation by distance, 2) morphological evolution was not influenced by climate, 3) but there may be a weak association of morphology and geography suggesting, as for the genetic data, a scenario of drift rather than adaptation.

## 4 DISCUSSION

### 4.1 Species delimitation and phylogenetic relationships

Phylogenetic (AFLP and mtDNA) and genetic structure analyses suggested that *T. sacchii*,* T*. cf. *chudeaui,* and *Theba* sp. 3 are genetically distinct entities (Figures 2 and 3). This result was in line with ecological niche analysis which indicated that these taxa are ecologically differentiated as well (Figures 6 and Figure S4). The results of genetic, morphological, and environmental niche analyses further subdivided *Theba* sp. 3 in at least two subgroups (Figures 2, 3, 4, 5 and Figures S2‐S4). Morphologically, a large northern and a small southern form can be distinguished (Figure 4). This distinction was paralleled by genetic subdivision and differentiation in realized niche space (Figures 2, 3 and Figure [Link], [Link]). As the number of samples of *Theba* sp. 3 large and *Theba* sp. 3 small was limited, the results of the environmental niche analyses have to be interpreted with caution. *Structure* analyses even identified three genetic clusters within *Theba* sp. 3 small in accordance with sampling sites (Figure 3).

Gene trees based on analyses of single or just a few genes can show incongruent topologies compared to species trees. Sources of genealogical discordance include locus‐specific effects such as convergence, selection, incomplete lineage sorting (ILS), introgressive hybridization, or horizontal gene transfer (HGT; Carstens & Knowles, 2007; Machado & Hey, 2003; Seehausen, 2004).

In the present study, the results of the phylogenetic analyses of COI and AFLP markers were inconsistent as already observed in analyses of the entire genus (Böckers, Greve, Hutterer, Misof, & Haase, 2016; Haase et al., 2014). In the Bayesian COI tree, *T. sacchii* and *Theba* sp. 3 were sister species with a high BPP of 95% (Figure 2b), whereas the AFLP distance tree placed the latter species into closer relationship to *T*. cf. *chudeaui* (Figure 2a). As the AFLP method allows a genome‐wide investigation of numerous markers, individual effects related to the particular evolutionary history of single loci are more likely to be compensated (Koblmüller et al., 2007; Sullivan, Lavoué, Arnegard, & Hopkins, 2004). We therefore expect that the AFLP NJ tree (Figure 2a) draws a more reliable picture of the evolutionary history of *T*. cf. *chudeaui*,* T. sacchii,* and *Theba* sp. 3 than the COI phylogeny (Figure 2b). Furthermore, in cases where species are parapatric advantageous alleles may readily spread across hybrid zones (Barton & Bengtsson, 1986). Such single gene introgressions seem especially common for cytoplasmically inherited genomes and in the past decades, discordant phylogenetic results between mitochondrial and nuclear gene markers were mostly attributed to adaptive introgression of mitochondrial DNA (Harrison, 1989; Rieseberg, 1995; Toews & Brelsford, 2012). Indeed, the highly supported sister group relationship in the COI topology (Figure 2b) along with the small environmental niche overlap between *T. sacchii* and *Theba* sp. 3 (3PCs: 0.037; 4PCs: 0.008; Figure S4) might indicate adaptive introgression of a mitochondrial COI variant favored in the partially shared niche. The short branch lengths of the COI topology (Figure 2b) indicate that differentiation processes among these taxa are fairly young and ILS of mitochondrial COI cannot be ruled out, however (Joly, McLenachan, & Lockhart, 2009).

### 4.2 Species divergence

The predominant mode of differentiation in land snails is most likely allopatric, nonadaptive speciation (Cook, 2008; Davison, 2002; Davison & Clarke, 2000; Goodacre, 2001; Greve et al., 2012; Holland & Hadfield, 2004; Sauer et al., 2013; Scheel & Hausdorf, 2012). Generally, genetic drift is rarely considered as sole driver of speciation. It is the combination of drift and selection that predominantly drives species divergence (Marie Curie Speciation Network, 2012). The present study provides a potential example, in which genetic drift is apparently the main driver of species differentiation.

The results of the environmental niche analyses (Figures 6 and Figure S4) suggested that all *Theba* taxa are ecologically well differentiated occupying realized niche spaces with no or low overlap. Interestingly, the environmental niche analysis suggested potentially suitable niche space for *T*. *sacchii*,* T*. cf. *chudeaui* and *Theba* sp. 3 small and *Theba* sp. 3 large along the coastal areas of the islands of Fuerteventura and except for *T*. cf. *chudeaui* also Lanzarote (Figures 6 and Figure S3). This might indicate that past colonization events of the islands or the mainland have probably been facilitated by already preadapted *Theba* species. Differentiation in environmental niches was predominantly explained by precipitation‐related variables (first and second PC) and, to a lesser extent, by variation in temperature‐related variables (third and fourth PC; Table S2). The results of the environmental niche analyses (Figures 6 and [Link], [Link]) may thus indicate that precipitation‐related variables play an essential role on the distribution patterns of these four desert taxa and on their ability to cope with desiccation. They may further suggest that climate‐associated selection might have played a role in shaping species diversity of *Theba* along the Atlantic coasts of South Morocco and Western Sahara. However, environmental niche analysis unsupported by other data is not sufficient to claim selection. Thus, we conducted comparative analyses in terms of Mantel and partial Mantel tests, despite being aware of their limitations (Guillot & Rousset, 2013). These results provided evidence for IBD for both genetic data and differentiation of shell shape indicating that neutral genetic differentiation among these forms could be best explained by limited migration and genetic drift. The distribution ranges of single species might have been disrupted by climate oscillations between humid and dry conditions in NW Africa beginning about 5–6 million years ago (Anhuf, 2000; DeMenocal, 2004; Rognon, 1993) promoting nonadaptive differentiation by geographic isolation. The fossil record of three *Theba* species collected from a Pleistocene dune in SW Morocco near Tan Tan Plage, for example, may indeed document the occurrence of range shifts through time: Fossil shells (in the present analysis represented by “Fossil”), most similar to *Theba* sp. 3 small and *T*. cf. *chudeaui*, indicated the occurrence of this complex of forms further north than today, *T. subdentata* extended much further south, and *T. tantanensis* probably became extinct in SW Morocco (Hutterer et al., 2010).

However, we cannot rule out that other selective forces than considered environmental variables may have played a role in genetic and morphological differentiation, but based on our results, the most parsimonious explanation is that nonadaptive speciation, that is, genetic drift, has probably been the predominant force driving divergence in *Theba* along the coast of South Morocco and the Western Sahara.

## CONFLICT OF INTEREST

None declared.
