# Supplementary figures and images for: Snails in the desert: Species diversification of Theba (Gastropoda: Helicidae) along the Atlantic coast of NW Africa
Source: Ecol Evol. 2017 Jun 22;7(14):5524–38. doi: 10.1002/ece3.3138 (PMC5528248; doi:10.1002/ece3.3138)

A

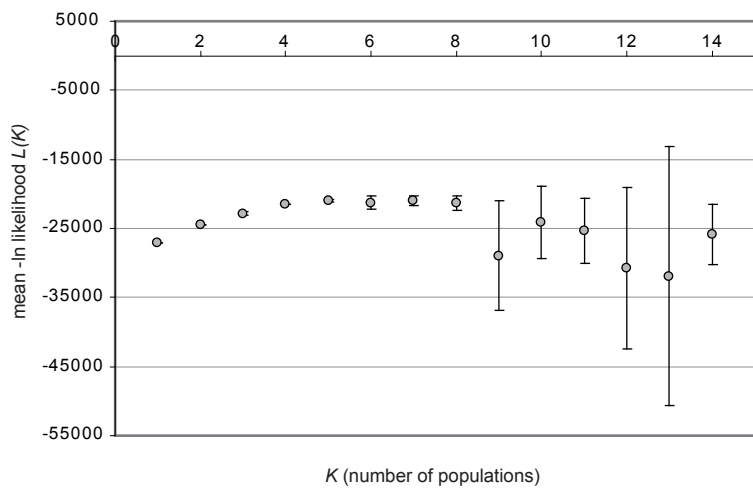

B

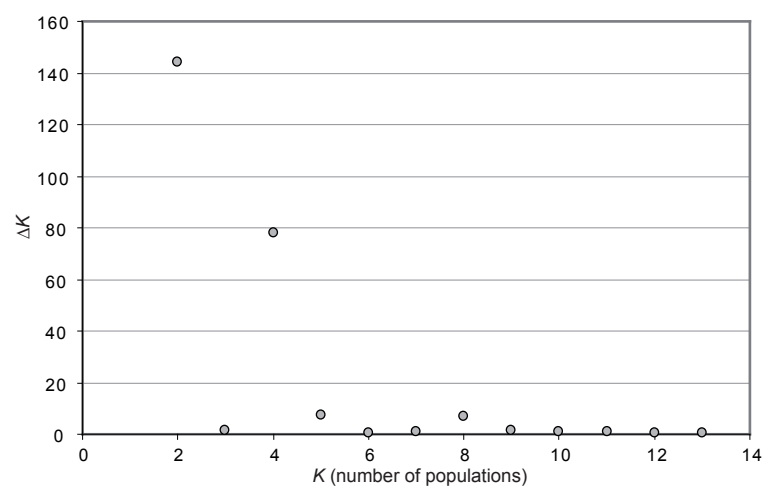

Supplement: Supplementary file 1 [file ECE3-7-5524-s001.pdf]

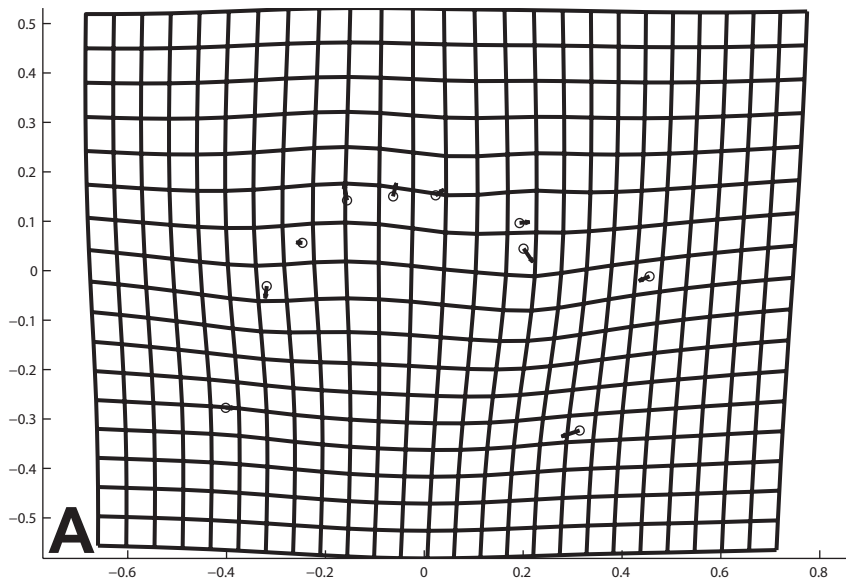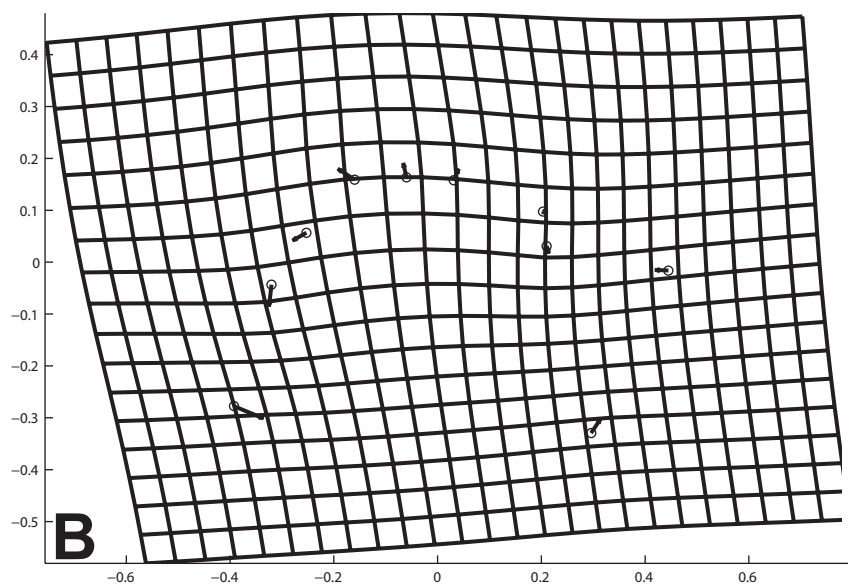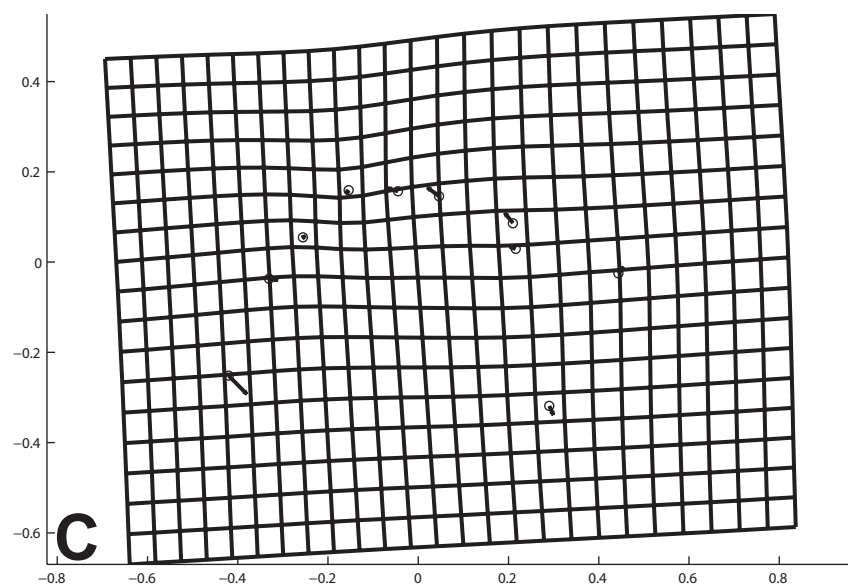

Supplement: Supplementary file 2 [file ECE3-7-5524-s002.pdf]

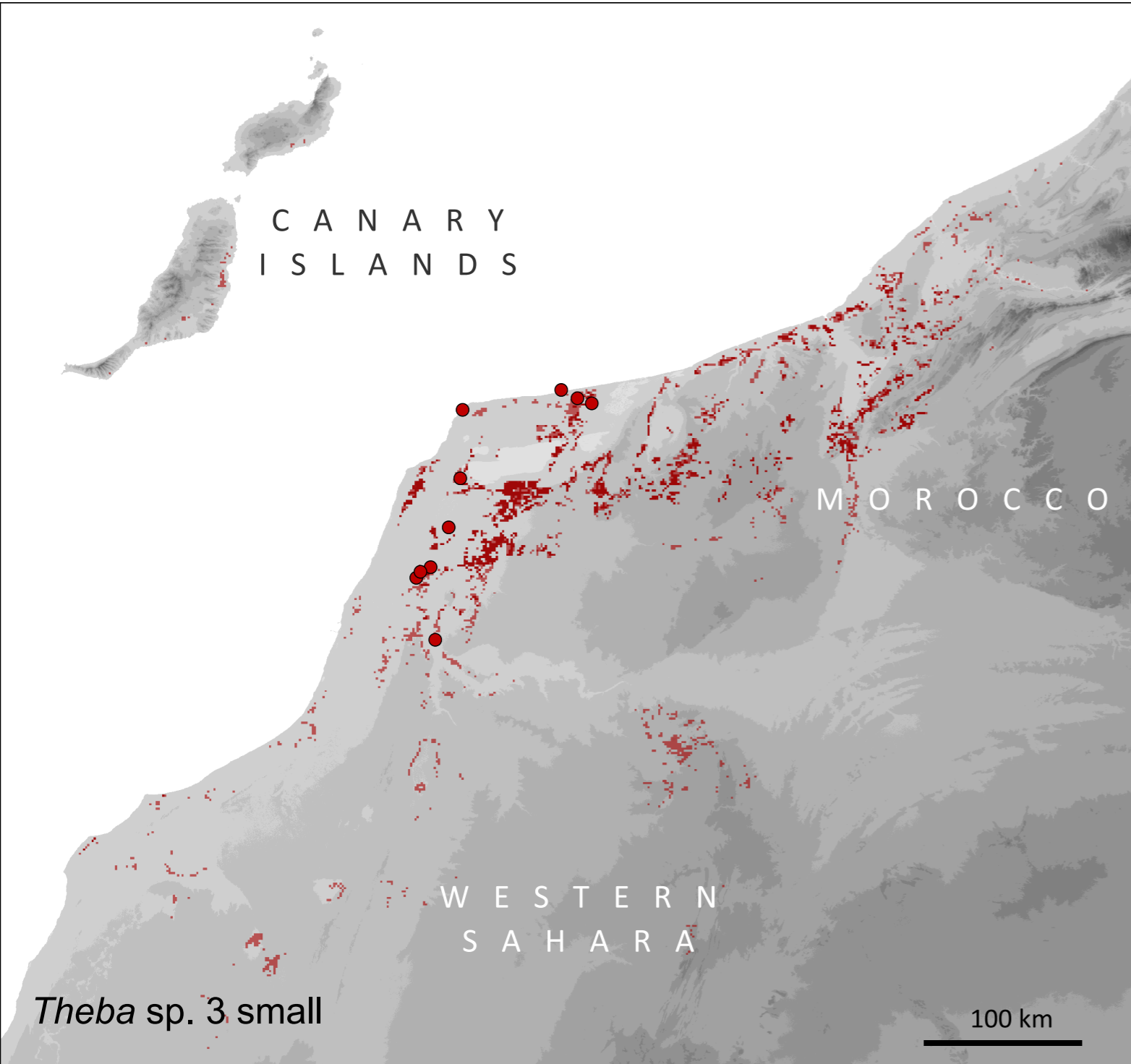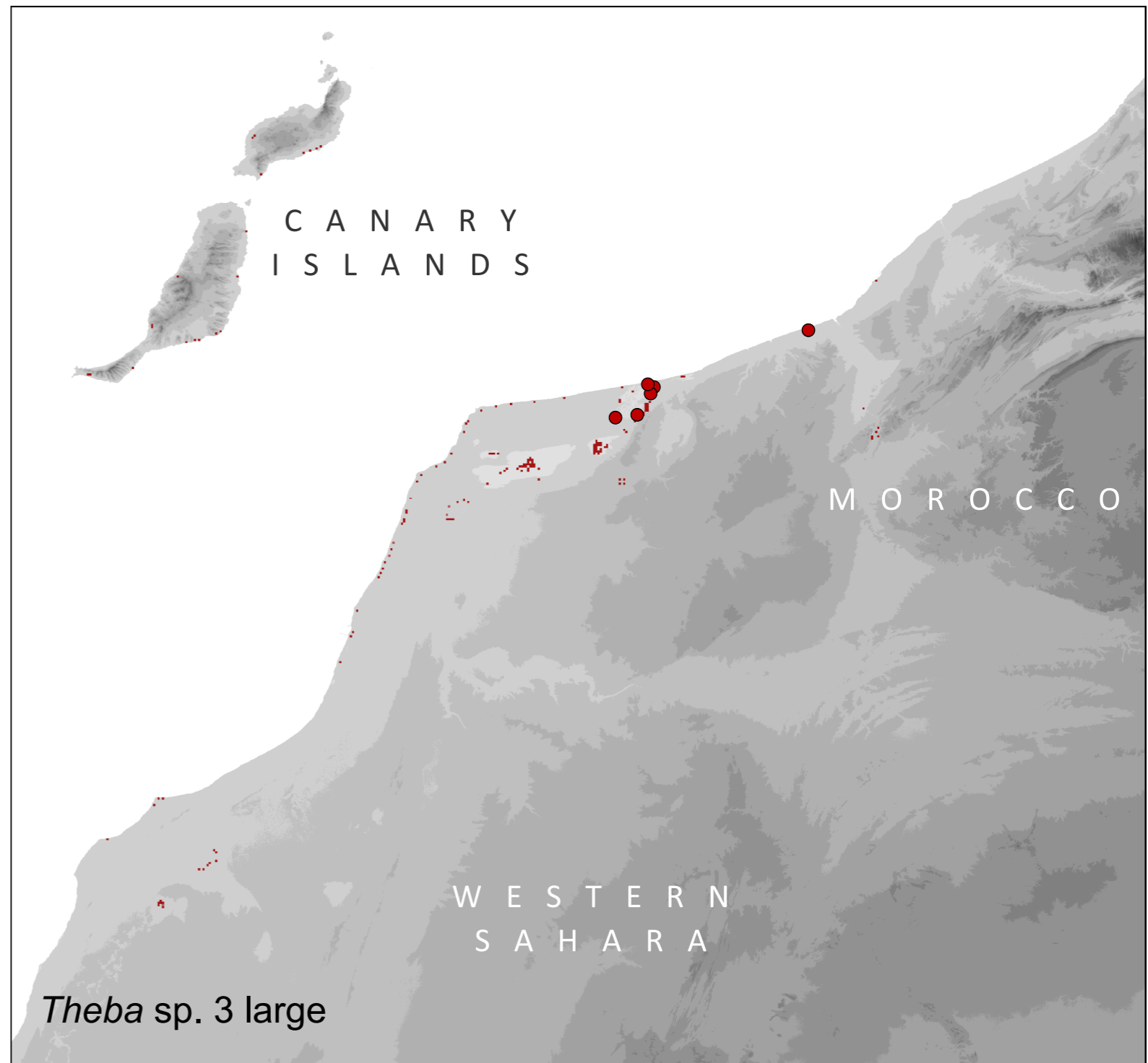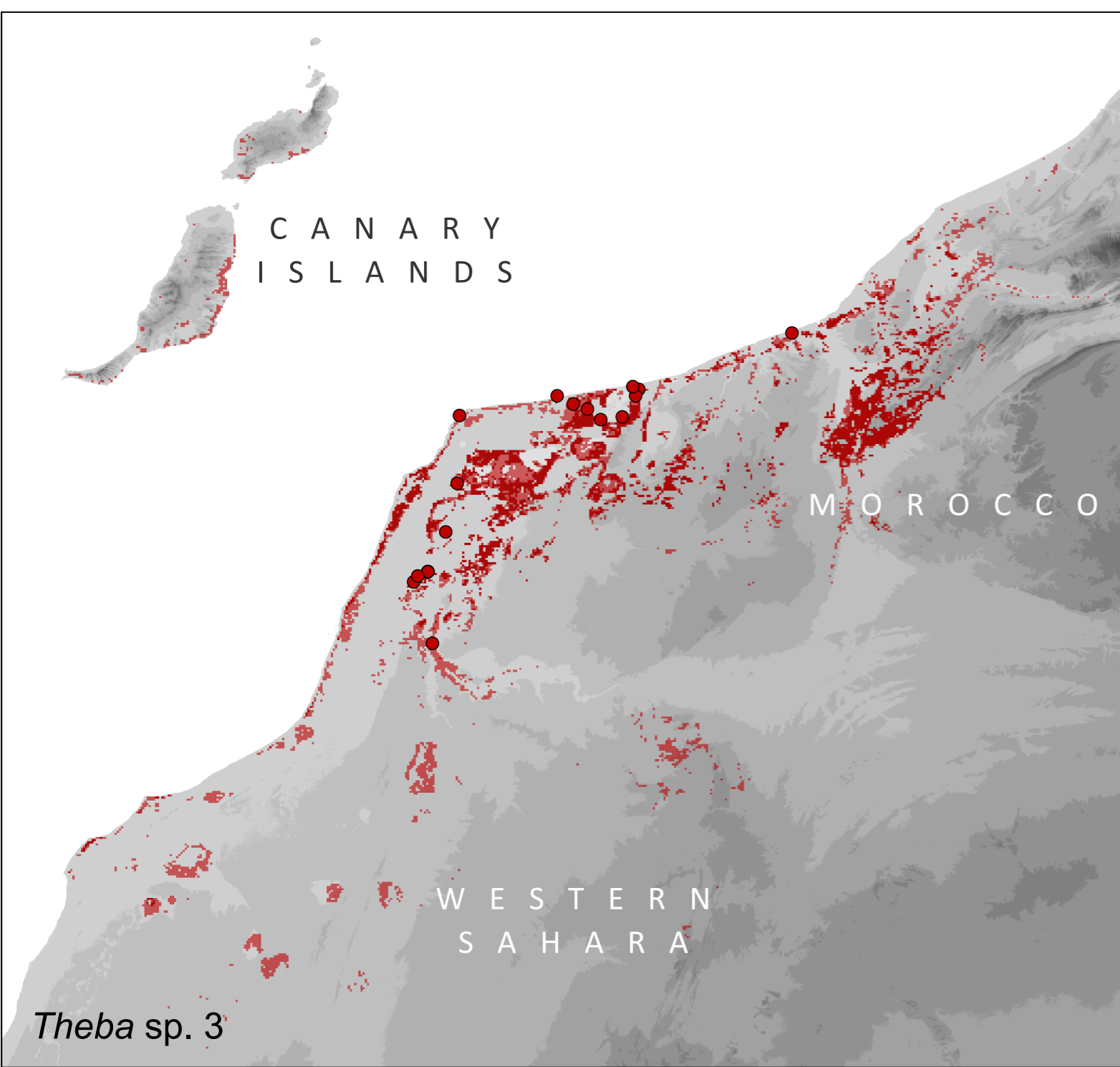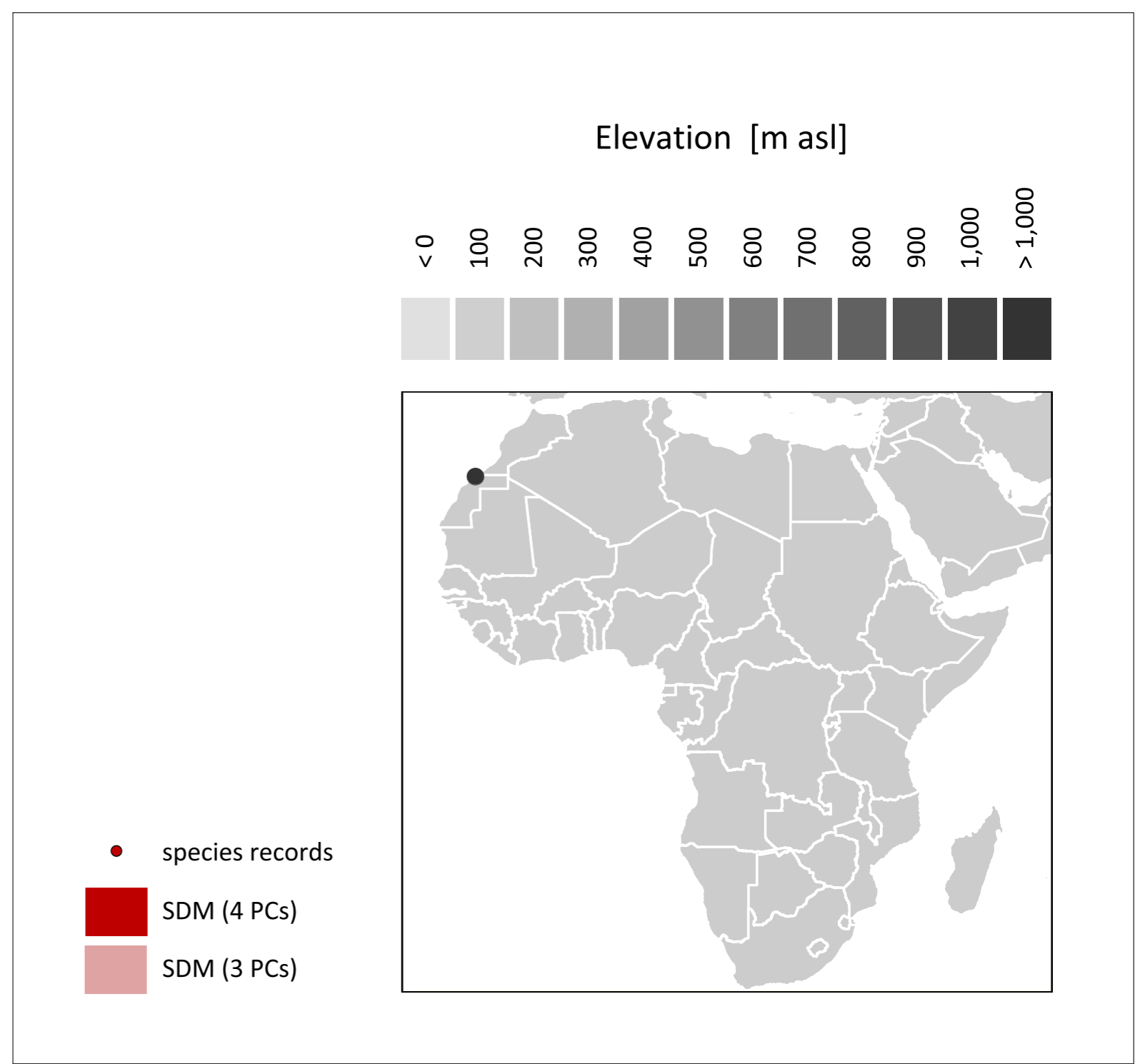

Supplement: Supplementary file 3 [file ECE3-7-5524-s003.pdf]

- ⋯ *Theba* sp. 3
- *Theba* sp. 3 small
- *Theba* sp. 3 large
- *Theba* cf. 3 chudeaui
- *Theba* sacchii

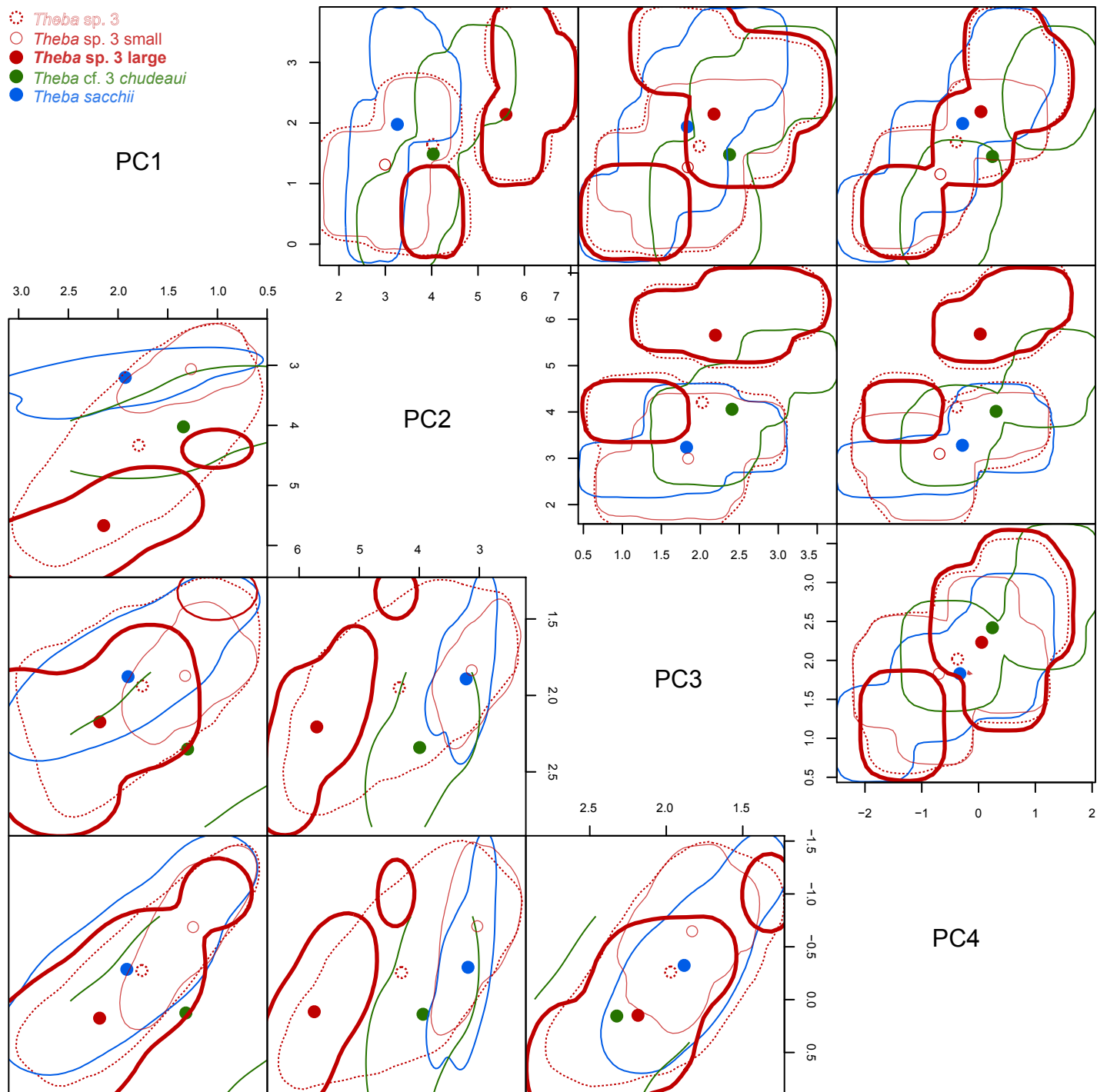

Supplement: Supplementary file 4 [file ECE3-7-5524-s004.pdf]
