# Supplementary material for: Snails in the desert: Species diversification of Theba (Gastropoda: Helicidae) along the Atlantic coast of NW Africa
Source: Ecol Evol. 2017 Jun 22;7(14):5524–38. doi: 10.1002/ece3.3138 (PMC5528248; doi:10.1002/ece3.3138)
Supplement: Supplementary file 5 [file ECE3-7-5524-s005.pdf]

Table S1. Detailed sampling information on specimens used in the present study.

Species	Code	Locality	Latitude	Longitude	# of specimens genotyped (AFLP)	# of specimens used in morphometrics	Collection†	COI GenBank Accession #
Theba subdentata	M24	Morocco, 12 km N Tiznit	N29°48.071'	W09°38.078'	3	-	ZFMK	HM034526, HM034528, KC526931
Theba subdentata	M55	Morocco, Oued Massa N. P.	N30°03.544'	W09°39.123'	2	-	ZFMK	HM034529
Theba solimae	M25	Morocco, 30 km N Bou Izakar	N29°26.766'	W09°41.688'	3	9	ZFMK	HM034494, KC526932-526933
Theba solimae	M27	Morocco, 1.6 km S Oued Draa	N28°30.983'	W10°57.443'	1		ZFMK	HM034495
Theba solimae	M59	Morocco, km 11 of road Bou Izakar-Goulimime	N29°07.456'	W09°48.156'	-	6	ZFMK	-
Theba solimae	M60	Morocco, Prov. Agadir, between Bou Izakar and Goulimime, 26 km NE of Goulimime	N29°06.552'	W09°49.975'	-	9	RMNH	-
Theba sp. 3 large	M28	Morocco, S Lagune de Khnifiss	N27°54.566'	W12°23.311'	11	20	ZFMK	HM034473-034474, KC526941
Theba sp. 3 large	M36	Morocco, 131 km S Tan Tan, 80 km E Tarfaya, few km N Lagune de Khnifiss	N27°55.271'	W12°18.169'	2	35	ZFMK	HM034480
Theba sp. 3 large	M69	Morocco, S Oueda Ma Fatma	N28°15.047'	W11°38.097'	-	4	ZFMK	-
Theba sp. 3 large	M70	Morocco, Prov. Laayoune, Guelta El Aouina (= Sidi Lemsid) near Puerto Cansado = 67 km E of Tarfaya	N28°01.727'	W12°14.352'	-	14	RMNH	-

Theba sp. 3 large	M71	Morocco, Prov. Laayoune, Lagune de Khnifiss = Puerto Cansado, 64 km east of Tarfaya	N28°00.173'	W12°15.100'	-	19	RMNH	-
Theba sp. 3 large	M72	Morocco, Prov. Laayoune, N-border of Sebkha Tazra (= Lagune de Khnifiss)	N28°02.335'	W12°15.672'	-	8	RMNH	-
Theba sp. 3 small	M33/48	Western Sahara, Dchira	N27°01.717'	W13°03.10 6'	3	10	ZFMK	HM034483, KC526942
Theba sp. 3 small	M34	Western Sahara, 69 km S Tarfaya	N27°18.702'	W13 °04.194'	9	6	ZFMK	HM034475-034477, KC526943
Theba sp. 3 small	M35/37/56	Western Sahara, 76 km S Tarfaya	N27°16.263'	W13°07. 498'	21	5	ZFMK	HM034478-034479, HM034481-034482, HM034484, KC526944-526947
Theba sp. 3 small	M73	Morocco, Prov. Laayoune, W of dune row between Sebkha Tazra and Sebkha Houiselgua = 47 km E of Tarfaya	N27°57.069'	W12°26.419'	-	5	RMNH	-
Theba sp. 3 small	M74	Morocco, Prov. Laayoune, desert region 2 km from the coast, 40 km E of Tarfaya, turn left at km sign Tan-Tan 189 in N direction	N27°58.224'	W12°29.792'	-	15	RMNH	-
Theba sp. 3 small	M75	Morocco, 37 km E Tarfaya, 174 km S Tan Tan	N28°00.246'	W12°33.573'	-	15	ZFMK	-
Theba sp. 3 small	M76	Morocco, Prov. Laayoune, central dunes W of Tarfaya village	N27°55.520'	W12°56.728'	-	17	RMNH	-
Theba sp. 3 small	M77	Morocco, Daora	N27°28.088'	W12°59.96'	-	19	ZFM K	-

Theba sp. 3 small	M78	Morocco, Tarfaya, 2 km S Tah	N27°39.55'	W12°57. 249'	-	14	ZFMK	-
Theba sp. 3 small	M79	Morocco, Prov. Laayoune, 14 km N of Laayoune along the main road to Tarfaya	N27°17.658'	W13°06.573'	-	3	RMNH	-
Theba sacchii	M39	Morocco, S Tan Tan, 36 km S of road from Tan Tan - Tan Tan Plage to Es Semara	N28°08.679'	W11°15.814'	14	13	ZFMK	HM034472, KC526 937-526940
Theba sacchii	M61	Morocco, Prov. Tarfaya, 7 km SW of Oued Draa, 2.4.1987	N28°29.365'	W11°00.238'	-	9	RMNH	-
Theba sacchii (Holotype)	M62	Morocco, Prov. Tarfaya, Goulimime-Tan Tan, 1.5 km SW Oued Draa	N28°31.077'	W10°57.275'	-	1	RMNH	-
Theba sacchii	M63	Morocco, 9 km N Tan Tan, in fossil sands	N28°28.605'	W11°00.965'	-	18	ZFMK	-
Theba sacchii	M64	Morocco, Tan Tan	N28°26.138'	W11°05.989'	-	2	ZF MK	-
*Theba sacchii**	-	Morocco, 5 km NW of Tan Tan	N28°26.691'	W11°03.07 6'	-	-	-	-
*Theba sacchii**	-	Morocco, 0.5 km NW of Tan Tan	N28°26.265'	W11°04. 043'	-	-	-	-
*Theba sacchii**	-	Morocco, Goulimime - Tan Tan, 12.5 km SW of the Oued Draa	N28°27.451'	W11°02.026'	-	-	-	-
Theba chudeaui (Syn- and Topotypes)	M82	Mauritania, Cap Blanc (= Ras Nouadhibou)	N20°46.283'	W17°02.833'	-	19	MNHN	-
Theba cf. *chudeaui*	M29	Western Sahara, 42 km S Laayoune	N26°51.394'	W1 3°28.168'	1	13	ZFMK	HM034485
Theba cf *chudeaui*	M30	Western Sahara, Laayoune Plage N	N27°06.723'	W1 3°23.878'	4	6	ZFMK	HM034486-034487

Theba cf. *chudeaui*	M31	Western Sahara, S El Marssa, 62 km S Laayoune	N26°41.451'	W13°33.116'	1	21	ZFMK	HM034488
Theba cf. *chudeaui*	M32	Western Sahara, 72 km S Laayoune	N26°39.180'	W1 3°38.775'	10	14	ZFMK	HM034489-034491, KC526934-5269 36
Theba cf. *chudeaui*	M80	Morocco, Prov. Laayoune, Laayoune plage (Cobeza playa)	N27°11.805'	W13°23.084'	-	2	RMNH	-
Theba cf. *chudeaui*	M81	Western Sahara, 52 km S Laayoune	N26°52.752'	W1 3°28.367'	-	7	ZFMK	-
Fossil	M65	Morocco, 2 km W Tan Tan, fossil sands along road	N28°27.569'	W11°07.195'	-	12	ZFMK	-
Fossil	M66	Morocco, Tan Tan plage, fossil dune 30 m from shore	N28°30.531'	W11°19.583'	-	32	ZFMK	-
Fossil	M67	Morocco, 6 km N Tarfaya, fossil dunes	N27°57.235'	W12°51.726'	-	34	ZFMK	-
Fossil	M68	Morocco, Amigrouw Plage, fossil dunes	N27°40.619'	W13°09.855'	-	5	ZFMK	-

* Records of species, which were only used for environmental niche analyses (Gittenberger & Ripken 1987).

† Abbreviations (Collection): ZFMK = Zoologisches Forschungsmuseum Alexander Koenig, Bonn, Germany; MNHN = Museum National d`Histoire Naturelles, Paris, France; RMNH = National Museum of Natural History (formerly Rijksmuseum van Natuurlijke Historie), Leiden, The Netherlands.
