# Supplementary material for: Snails in the desert: Species diversification of Theba (Gastropoda: Helicidae) along the Atlantic coast of NW Africa
Source: Ecol Evol. 2017 Jun 22;7(14):5524–38. doi: 10.1002/ece3.3138 (PMC5528248; doi:10.1002/ece3.3138)
Supplement: Supplementary file 6 [file ECE3-7-5524-s006.pdf]

Table S2. Environmental variables and derived variable sets used to build hypervolume models. Loadings for the first four principal components with an eigenvalue >1.

ID	Remote sensing variable	Derived variable	PC1	PC2	PC3	PC4
Bio 1	-	Annual mean temperature	-0.812	-0.33	0.19	0.276
Bio 7	-	Temperature annual range	-0.228	-0.445	-0.732	0.394
Bio 10	-	Mean temperature of warmest quarter	-0.717	-0.453	-0.2	0.39
Bio 11	-	Mean temperature of coldest quarter	-0.725	-0.072	0.606	0.071
Bio 12	-	Annual precipitation	0.898	0.024	-0.217	-0.206
Bio 16	-	Precipitation of wettest quarter	0.903	0	-0.16	-0.221
Bio 17	-	Precipitation of driest quarter	0.746	0.222	-0.354	-0.101
ED1503_Bio1	Near-Infrared	Annual mean Near-Infrared	-0.811	-0.513	-0.058	-0.221
ED1503_Bio7	Near-Infrared	Near-Infrared annual range	0.436	-0.264	-0.793	-0.133
ED1503_Bio10	Near-Infrared	Mean Near-Infrared of highest quarter	-0.617	-0.613	-0.386	-0.269
ED1503_Bio11	Near-Infrared	Mean Near-Infrared of lowest quarter	-0.847	-0.249	0.372	-0.109
ED150708_Bio1	MODIS V4 Band 07 + 08 Synoptic months: / day- + night-time land surface temperature	Annual mean temperature (remote)	-0.789	-0.504	0.06	-0.305
ED150708_Bio7	MODIS V4 Band 07 + 08 Synoptic months: / day- + night-time land surface temperature	Temperature annual range (remote)	-0.103	-0.475	-0.819	0.152

ED150708_Bio10	MODIS V4 Band 07 + 08 Synoptic months: day- + night-time land surface temperature	Mean temperature of warmest quarter (remote)	-0.678	-0.608	-0.248	-0.262
ED150708_Bio11	MODIS V4 Band 07 + 08 Synoptic months: day- + night-time land surface temperature	Mean temperature of coldest quarter (remote)	-0.748	-0.207	0.509	-0.333
ED1514_Bio1	MODIS V4 Band 14 Synoptic months: Normalised Difference Vegetation Index (NDVI)	Annual mean NDVI	0.815	-0.501	0.216	0.076
ED1514_Bio7	MODIS V4 Band 14 Synoptic months: Normalised Difference Vegetation Index (NDVI)	NDVI annual range	0.766	-0.359	0.078	-0.284
ED1514_Bio10	MODIS V4 Band 14 Synoptic months: Normalised Difference Vegetation Index (NDVI)	Mean NDVI of highest quarter	0.833	-0.495	0.198	-0.035
ED1514_Bio11	MODIS V4 Band 14 Synoptic months: Normalised Difference Vegetation Index (NDVI)	Mean NDVI of lowest quarter	0.754	-0.513	0.243	0.155

ED1515_Bio1	MODIS V4 Band 15 Synoptic months: Enhanced Vegetation Index (EVI)	Annual mean EVI	0.71	-0.53	0.392	0.174
ED1515_Bio7	MODIS V4 Band 15 Synoptic months: Enhanced Vegetation Index (EVI)	EVI annual range	0.747	-0.368	0.078	-0.249
ED1515_Bio10	MODIS V4 Band 15 Synoptic months: Enhanced Vegetation Index (EVI)	Mean EVI of highest quarter	0.755	-0.531	0.339	0.053
ED1515_Bio11	MODIS V4 Band 15 Synoptic months: Enhanced Vegetation Index (EVI)	Mean EVI of lowest quarter	0.581	-0.523	0.454	0.284
Eigenvalues			12.008	4.088	3.723	1.223
Explained variance			52.211	17.775	16.188	5.318
